# The multifaceted role of the SASP in atherosclerosis: from mechanisms to therapeutic opportunities

**DOI:** 10.1186/s13578-022-00815-5

**Published:** 2022-05-31

**Authors:** Yu Sun, Xia Wang, Tianwei Liu, Xiaoyan Zhu, Xudong Pan

**Affiliations:** 1 Department of Neurology, The Affiliated Hospital of Qingdao University, Qingdao, Shandong China; 2 Institute of Cerebrovascular Diseases, The Affiliated Hospital of Qingdao University, Qingdao, Shandong China; 3 Department of Critical Care Medicine, The Affiliated Hospital of Qingdao University, Qingdao, Shandong China

**Keywords:** SASP, Atherosclerosis, Senescent cell, Inflammation, Ageing

## Abstract

**Background:**

The global population of older individuals is growing, and ageing is a key risk factor for atherosclerotic cardiovascular diseases. Abnormal accumulation of senescent cells can cause potentially deleterious effects on the organism with age. As a vital marker of cellular senescence, the senescence-associated secretory phenotype (SASP) is a novel mechanism to link cellular senescence with atherosclerosis.

**Main body:**

In this review, we concretely describe the characteristics of the SASP and its regulation mechanisms. Importantly, we provide novel perspectives on how the SASP can promote atherosclerosis. The SASP from different types of senescent cells have vital roles in atherosclerosis progression. As a significant mediator of the harmful effects of senescent cells, it can play a pro-atherogenic role by producing inflammation and immune dysfunction. Furthermore, the SASP can deliver senescence signals to the surrounding vascular cells, gradually contributing to the development of atherosclerosis. Finally, we focus on a variety of novel therapeutic strategies aimed to reduce the burden of atherosclerosis in elderly individuals by targeting senescent cells and inhibiting the regulatory mechanisms of the SASP.

**Conclusion:**

This review systematically summarizes the multiple roles of the SASP in atherosclerosis and can contribute to the exploration of new therapeutic opportunities.

## Introduction

Ageing has long been considered a major risk factor for many chronic and fatal diseases [1], including cardiovascular diseases (CVDs) [2], neurodegenerative diseases and cancer [3]. Atherosclerosis as a chronic inflammatory disease of the blood vessel walls is the main potential cause of CVD [4]. It is well known that there are many different risk factors for atherosclerosis such as high blood pressure, low-density lipoprotein (LDL) cholesterol, diabetes and smoking [5]. At the same time, increasing evidence suggests that ageing is one of the greatest risk factors for atherosclerosis [6]. Because ageing is a modifiable factor, it can be viable to slow down the development of age-related diseases by regulating basic ageing mechanisms. One of the mechanisms is cell senescence, which can lead to chronic inflammation through the SASP [7].

The SASP contributes to the secretion of inflammatory cell cytokines and chemokines that induce local and systemic inflammatory responses, immune system activation, tissue damage and fibrosis, and cell apoptosis and dysfunction. Moreover, the SASP can also induce the enlargement of local and systemic senescence to neighbouring cells via paracrine or endocrine mechanisms [8]. Furthermore, a variety of molecules involved in the SASP can serve as promoters and biomarkers of cardiovascular diseases including atherosclerosis [9]. Recent clinical trials have clearly demonstrated a causal relationship between inflammation and human atherosclerosis [10]. Atherosclerosis is considered a chronic inflammatory disease, and atherosclerotic plaques present with cell senescence [11]. Cell senescen ce and atherosclerosis have multiple common aetiological stimuli, but senescent cells are not just simple bystanders. Senescent cells from atherosclerotic plaques are lack proliferation, overexpress P16INK4A, P53, P21 [12–14], and increase the activity of senescence-associated beta-galactosidase (SAβG) [15]. They can also establish the SASP, which can cause increased secretion of various inflammatory cell cytokines, chemokines and matrix-degrading proteases [16]. Notably, there is evidence that the SASP, as a source of chronic inflammation and some plaque instability factors, is involved in the pathogenesis and development of atherosclerosis [17].

The SASP from senescent cells exerts many pro-atherogenic effects, which may involve vascular remodelling, plaque formation and rupture. There is evidence that plaque-rich arteries contain various typical SASP components, including matrix metalloproteinases and multiple inflammatory factors. However, these phenomena are not present in normal adjacent blood vessels [17]. Senescent cells in blood vessels with the SASP release various inflammatory cytokines (interleukin-6 and interleukin-8) and growth factors (such as VEGF, PDGF, chemokines and MMPs) [18]. Studies have shown that some of these are known cardiovascular risk factors. Additionally, a study reported that p16 positive cells are the main driver of the aged heart phenotype that causes a reduced lifespan in mice [19], so removing senescent cells with p16 promoter activity can inhibit the occurrence and development of atherosclerotic plaques and improve the stability of plaques [17, 20].

In this review, we attentively summarized the relevant characteristics and regulatory mechanisms of the SASP. Moreover, we describe the SASP as the hallmark of senescence and SASP-related extracellular vesicles (EVs) and how it plays an important role in senescence transmission via paracrine or endocrine signalling. Furthermore, we focused on the multifaceted role of the SASP, as an important common feature of various types of senescent cells, on the progression of atherosclerosis and the value of the SASP as a pathological target and treatment for atherosclerosis.

## Definition and composition of the SASP

General cognitive and additional evidence indicate that human body function damage is usually caused by cell senescence or organ senescence. Senescent cells usually show increased lysosomal β-galactosidase activity and secrete a set of powerful inflammatory cytokines known as the SASP [21, 22]. The SASP is a condition first identified by Coppe and his colleagues, in which senescent cells secrete some substances that contribute to a range of physiological or pathological effects [23]. Interestingly, the SASP varies from senescent cells to senescent cells according to the cell type of senescent cells, induction of senescent cells, the time after induction, and how they respond to hormones, drugs, and various other factors [24]. The various biological functions induced by each component of the SASP suggested that it might contact local and neighbouring cells and constitute a mechanism for regulating the microenvironment. Additionally, whether it is harmful depends on secretory factors, cell type, duration and secretion-induced stimulation [25].

The SASP produces inflammatory cytokines such as IL-1a, IL-1b, IL-6, IL-8, IL-18, CCL-2, tumour necrosis factor (TNF-a), metalloproteases (MMP-1, -3, -8, -9, -13), and many growth factors, including vascular endothelial growth factor (VEGF), PDGF-AA [26] and other factors [17, 27]. Moreover, the SASP also consists of various proteins and nonprotein molecules, such as proteases, haemostatic factors, ceramides, bradykinin, extracellular matrix (ECM) components, and damage-associated molecular patterns (DAMPs) [28–30]. Other SASP components include vesicles, exosomes, various microRNAs and noncoding RNAs, certain fragments of DNA, some other nucleotides, ROS, prostaglandin analogues, protein aggregates, and other factors that transmit ageing signals and promote inflammatory responses [24, 31]. Notably, senescent cells with pro-inflammatory SASP can cause substantial pathological damage and lead to phenotypes associated with ageing [8].

**Fig.1:**
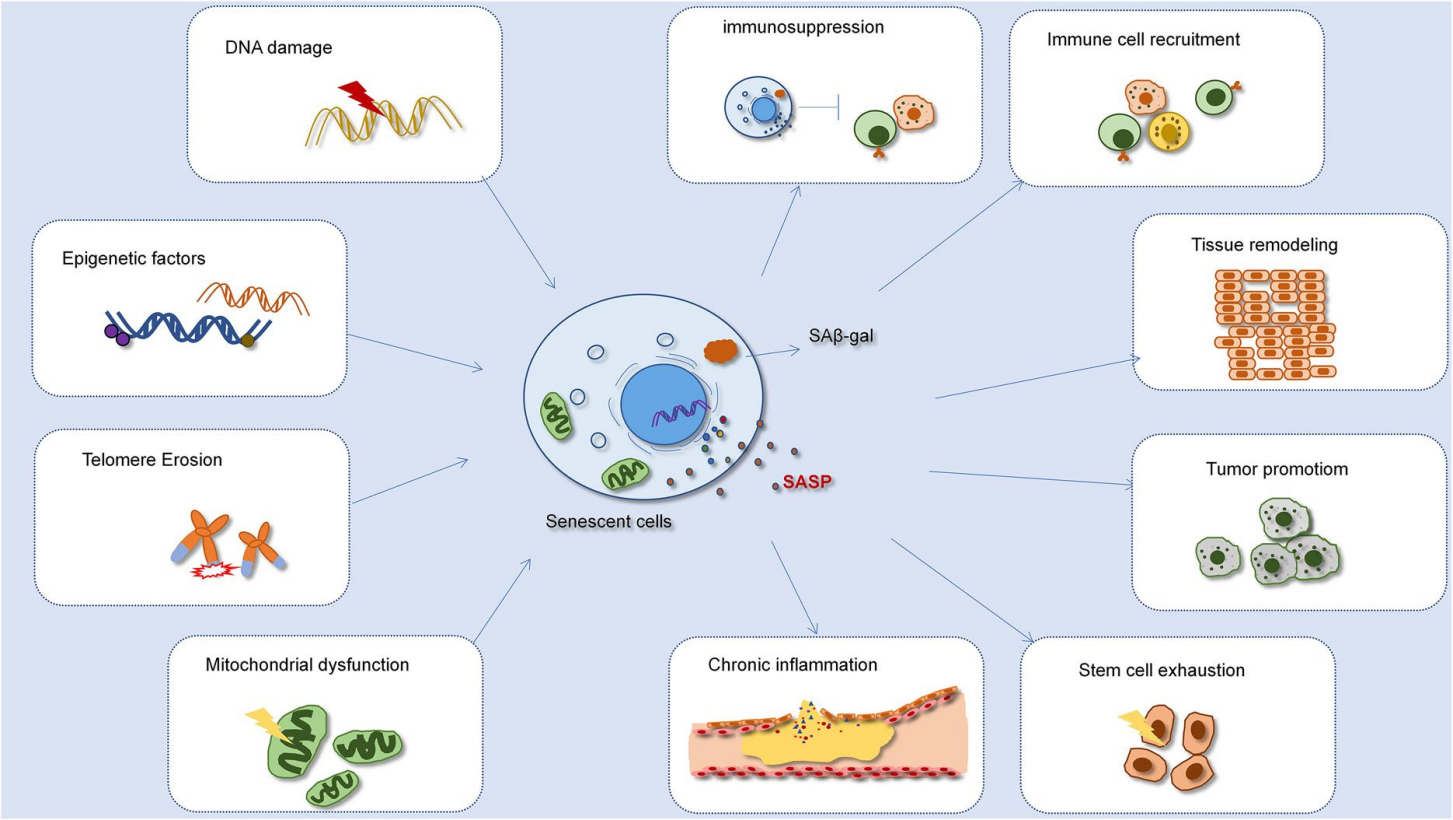
The multifaceted functions of the SASP

As a potential mechanism of senescence, the SASP plays a variety of roles such as enhancing ageing signals, influencing the tissue microenvironment around cells, and even affecting the whole body (Fig. 1) [32]. The SASP can cause dysfunction in multiple aged organs. For example, elevated levels of IL-6, IL-1 receptor antagonists and TNF receptor in the blood are important factors of SASP that predict chronic disease in the elderly [32]. More importantly, emerging experimental evidence suggests that the SASP is a source of long term and chronic inflammation and a cause of plaque instability that contributes gradually to the pathogenesis of atherosclerosis in comparison to acute inflammatory responses [9]. The various roles of SASP factors from senescent cells in atherosclerosis will be discussed in further detail.

Various internal and external stimuli lead to cell senescence. The SASP as a characteristic of senescent cells plays various roles through autocrine or paracrine. The SASP can recruit immune cells to eliminate senescent cell, but it also can lead to immunosuppression. Furthermore, the SASP can contribute to inflammation, tissue remodeling, and stem cell exhaustion.

## Regulatory mechanisms of the SASP

Several cellular regulatory factors such as p38MAPK, Jak2/Stat3, inflammasomes, m-TOR, miRNA, GATA-4, macroH2A1 and ATM have been proven to play roles during the generation of SASP. It is worth noting that the regulation of the SASP appears to be centred on the NF-kB complex [33] and C/EBP family [34, 35], both of which are involved in the regulation of cell stress and inflammatory signals. Their expression is active in senescent cells [36] and synergistically regulates the transcriptional mechanisms of the expression of different SASP components [37]. The production of the SASP cytokines depends largely on stress-induced NF-kB and p38MAPK signals [38–40], and is regulated by m-TOR dependent protein translation [41, 42].

**Fig.2:**
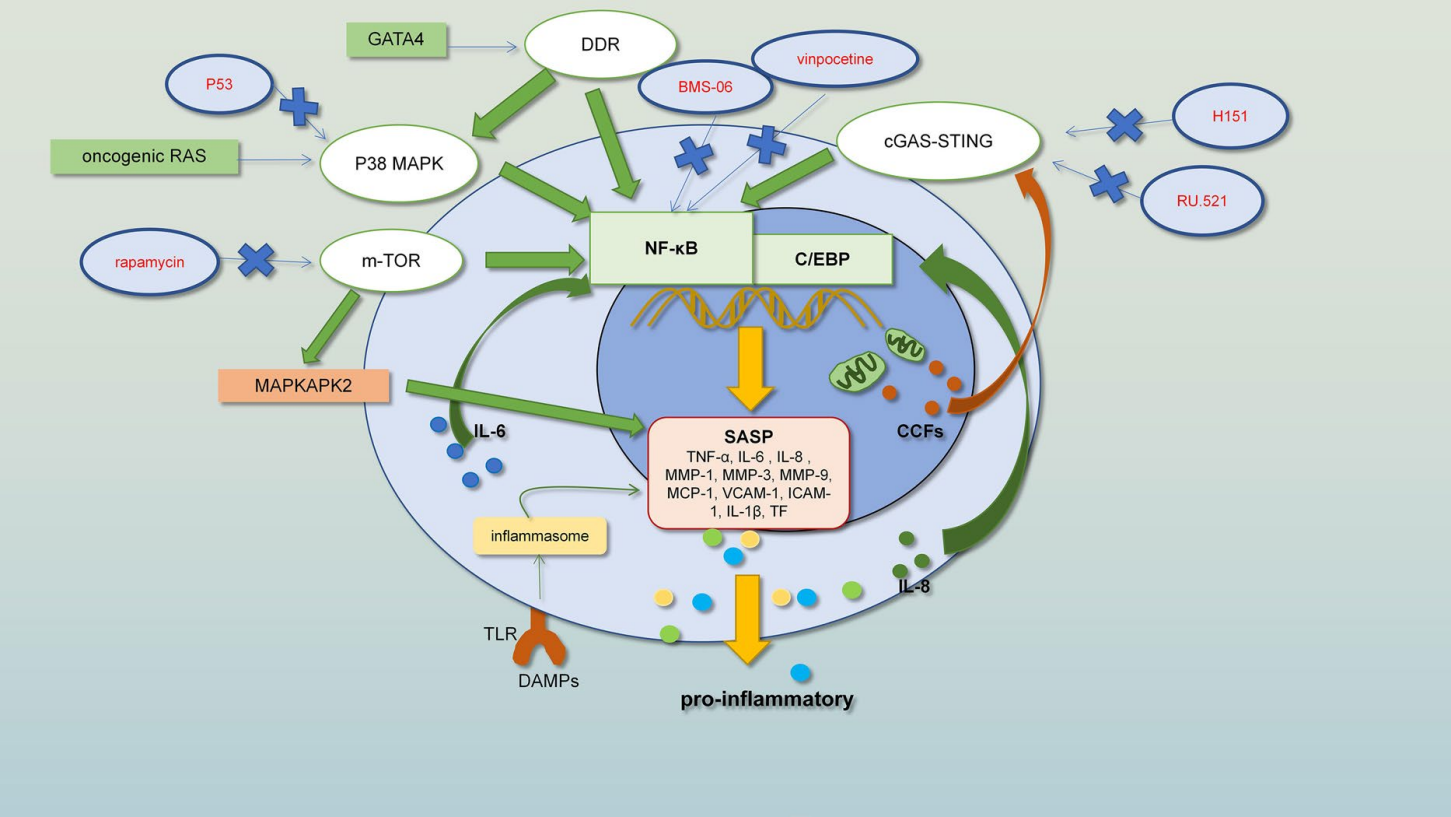
Regulatory mechanisms of the SASP

The associated secretory group and the SASP generate a self-sustaining intracellular signal cycle and inflammatory cascade involving the NF-κB, IL-1α, TGF-β, and IL-6 signalling pathways in age-related diseases. The main inflammatory factors, especially IL-6 [23, 43, 44], have significantly contributed to the inflammatory ageing phenomenon in healthy older people. The IL-6 trans-signalling pathway usually causes a chronic inflammatory response, increases adhesion molecules in the vascular system and increases vascular permeability [45]. Studies in mice show that senescence is associated with increased production of IL-6 in arteries, which take part in the positive feedback loop with mitochondrial dysfunction of the vasculature to promote the development of atherosclerosis [45]. Below, we summarize multiple regulatory pathways of the SASP (Fig. 2).

The images show various regulatory factors and regulatory pathways of the SASP. (The regulatory pathways are shown in green. Inhibitors that target these regulatory mechanisms are shown in red.)

## Through the NF-kB signalling pathway

Many studies have demonstrated that NF-kB [38, 46] and C/EBP [47] take part in the generation of the SASP in the progression of cell senescence. NF-κB and C/EBPβ are activated and enriched in the chromatin of senescent cells and regulate the composition of the SASP by regulating the transcription of IL-8 or IL-6, a key regulator of the inflammatory SASP. In turn, IL-6 and IL-8 enhance the activity of C/EBPβ and NF-κB through an autocrine feed-forwards loop and amplify the SASP signalling pathway [48]. C/EBPβ has been shown to regulate many factors including IL-1β, IL-8, IL-6, GROα and NAP2, which are part of the SASP [49]. Specifically, NF-kB positively regulates many genes encoding inflammatory cytokines and thus acts as the primary regulatory factor of the SASP [25]. The activity of interleukin-1α, interleukin-6, tumour necrosis factor and NF-κB, and other pro-inflammatory cytokines, is found to increase with age in tissues. There is also evidence that inhibiting NF-κB may provide some protection against progeria [50]. It has been reported that intracellular IL-1α/MIRI-146A/B/IL-6/C/EBP-β rings and p38/NF-κb associated and m-TOR mediated pathways contribute to altered gene expression of the SASP[7]. The IL-1α/IL-1R axis promotes the production of the SASP by the NF-κB signalling pathway, and regulation of the IL-1α/IL-1R axis can control NF-κB and inhibit its transcriptional activity [51].

GATA4 [40] connects autophagy and DDR with senescence and inflammation through activation of NF-kB, and then produces the SASP, including some inflammatory cytokines, possibly inducing paracrine senescence in normal cells [52, 53]. Furthermore, the NF-κB signalling pathway plays a vital role in driving endothelial cell inflammation and dysfunction by coordinating the expression of inflammatory cytokines and chemokines involved in atherosclerosis. Studies have shown that endothelial restriction inhibits NF-κB and reduces the formation of atherosclerotic plaques in ApoE ^− / −^ mice [54, 55], which indicates that inhibition of NF-κB associated pro-inflammatory gene expression in blood vessels can delay the development of atherosclerosis [56].

## Through the DNA damage reaction

Activation of the SASP is usually associated with the DNA damage response (DDR) [57, 58]. In short, DNA damage responses are induced by the recognition of faulty DNA. Recognition and signal transduction are achieved by recruiting a variety of sensor proteins such as P-ATM and gamma-H2AX. In vivo, the expression of P-ATM and γ-H2AX was positively correlated with the degree of atherosclerotic plaque formation. In addition, the expression of both proteins in plaque VSMCs is higher than that in normal vascular cells in vitro [59]. Recruitment of these proteins results in the activation of some effectors to take part in the repair of DNA. However, when DNA damage is too serious to recover, cell senescence and apoptosis occur [5]. The DNA damage response triggers cell senescence, which ultimately leads to the SASP.

During DNA damage-induced senescence, there is increasing evidence that the PIKK family includes a series of protein kinases that play a key role in initiating senescence and transcription of the SASP gene upstream of NF-kappa B signalling in response to DDR [60, 61]. The stability of the transcription factor GATA4 is enhanced, which binds DDR to the activation of NF-kappa B and thus induces the SASP [40]. Activated NF-kappa B is found in SMCs, macrophages and ECs of human atherosclerotic lesions. The activation of NF-kappa B in these cells controls the expression of the pro-inflammatory factors TNF-α, interleukin-6, -8, MMP-1, -3, -9 and tissue factor (TF) [62]. Notably, once activated, NF-kappa B translocates to the nucleus and increases the expression of inflammatory cytokines, including interleukin-6, interleukin-1β and tumour necrosis factor-α, chemokines such as MCP-1 and cell adhesion molecules including VCAM-1 and ICAM-1, which can lead to vascular damage and the development of atherosclerosis by increasing the generation of these inflammatory factors from the SASP [63–65].

## Through the p38MAPK signalling pathway

p38MAPK plays a vital role in the production of cell senescence and immune senescence [66, 67]. Activation of the TGF-β signalling pathway and p38MAPK are vital regulators of SASP states related to cellular and immune senescence [33, 39, 68]. P38, a component of the MAPK family, regulates SASP by activating NF-kappa B [39, 57]. We found that the activity of p38MAPK is important for the senescence-induced production of the SASP through direct DNA damage or oncogenic RAS. In these cases, p38MAPK is not rapidly activated but has delayed the kinetics of the SASP. In addition, p53 inhibits the SASP by inhibiting the activation of p38MAPK, which occurs independently of DDR. P38MAPK mainly regulates the SASP by NF-kB trans-inhibitory activity, which we found to be necessary for the production of many SASP factors. This study suggests that the p38MAPK pathway plays a new role in the regulation of senescence [39]. Moreover, p38 is a major regulator of DDR, and its activation induces the NF-kB signalling pathway and leads to the expression of SASP-related genes [25].

## Through the m-TOR pathway

Rapamycin complex 1(mTORC1), a lysosomal serine threonine kinase activated by senescent cells, regulates the physiological activity of proteins and cells and autophagy [69–72]. Induction of mTORC1 in senescent cells can lead to the development of the SASP, and mTORC1 inhibitors appear to decrease the inflammatory response caused by senescent cells and can also inhibit the SASP through different mechanisms [73, 74]. Furthermore, the m-TOR signalling pathway can control the activity of mitochondria to regulate energy metabolism and the progression of oxidative phosphorylation. The ROS-induced DDR can be activated by mTORC1-associated mitochondrial biogenesis through the ATM/Akt/mTORC1 phosphorylation cascade and then promote the regulation of PCG-1β, thereby affecting deteriorating senescence and the persistence of the SASP in vitro and in vivo [75].

Moreover, the m-TOR pathway in ageing can interfere with the metabolism of proteins, prevent autophagy and regeneration, and promote the senescence of cells and tissues [61]. M-TOR has also been reported as the primary regulator of the SASP. Rapamycin inhibits m-TOR to decrease the expression of IL-1α, resulting in a decrease in the transcriptional activity of NF-κB [42, 65]. MAPKAPK2, a kinase downstream of p38, can be regulated by m-TOR. Furthermore, it can phosphorylate and inhibit the RNA-binding protein ZFP36L1 during ageing and stabilize the transcription of the SASP mRNA [41]. Notably, inhibiting the m-TOR pathway is reported not only to be anti-ageing, but also to produce anti-atherosclerosis effects [76–79].

## Through the cGAS-STING signalling pathway

We recently found that fragments of cytoplasmic chromatin (CCFs) extruded from the nuclei of senescent cells trigger the SASP by activating the innate immune cytoplasmic DNA-sensing cGAS-STING pathway [80]. Cyclic GAS stimulating factor in the signalling pathway of interferon gene (sting) is a key marker of innate immune response that promotes the SASP to respond to the increase of cytoplasmic DNA (including the fragment of cytoplasmic chromatin, mitochondrial DNA and cDNA) from senescent cells [81]. In particular, we found that cGAS can recognize cytoplasmic chromatin fragments in senescent cells. The activation of cyclic GAS [82, 83], in turn, triggers the production of the SASP factors via stimulators (sting) of the interferon gene, thus contributing to paracrine senescence [82]. The cGAS-STING pathway is thought to be a vital regulatory factor for the induction of the SASP [82–84]. Furthermore, the cGAS/STING pathway contributes to activation of NF-κB signalling, which turns on the transcription of pro-inflammatory genes [82–84].

## Effect of the SASP on atherosclerosis through inflammation

Senescent cells have several external consequences, and the most important extrinsic factor is inflammatory responses [23, 85]. Chronic low-level inflammation is a serious complication of most diseases in which danger increases with age [86, 87]. Inflammatory markers such as IL-1 and IL-6 that operate as prognostic markers for illnesses such as atherosclerosis [88] confirm this negative effect of inflammation [85].

Senescent cells are involved in developing various diseases by releasing the SASP factors [89], which are responsible for transferring senescence to adjacent cells, thus leading to persistent low-level chronic inflammation, defined as inflammatory ageing. CANTOS has shown that targeting inflammation has a significant therapeutic effect on atherosclerotic diseases [90]. Furthermore, inflammatory ageing promotes the development of catastrophic atherosclerotic thrombotic complications by increasing platelet reactivity and predisposing patients to plaque rupture and erosion [91]. There is evidence that human atherosclerotic plaques contain cells with multiple senescence markers related to the severity of the diseases [89]. Cellular senescence is considered a vital source of the inflammatory response in atherosclerosis and CVDs [9].

Senescent cells present in atherosclerotic lesions from LdLR^ − / −^ mice fed a low-fat diet (LFD) have high transcription levels of p16Ink4a, and p19Arf. At the same time, various typical SASP components of the plaque-rich aortic arch, including matrix metalloproteinases MMP-3 and MMP-13, and inflammatory factors IL-1α and TNF-α, are increasing [17]. However, these phenomena are absent in normal adjacent vessels or aortas [17]. Senescent vascular cells are characterized by the SASP and release various inflammatory cytokine growth factors, chemokines and MMPs, some of which are known as cardiovascular risk factors [18]. Furthermore, senescent vascular cells exhibit the SASP that enables them to communicate with the microenvironment of other cells and promote senescent regeneration and embryonic development of adjacent cells and tissues [20].

Advanced atherosclerosis has a large number of cells with senescent characteristics that support the pro-inflammatory state, leading to the formation of necrotic cores, which ultimately lead to the fragility and rupture of the plaque, formation of thrombosis and acute vascular occlusion. This is because the expression of cells in advanced atherosclerotic plaques further aggravates the SASP of inflammation and simultaneously produces metalloproteases that degrade the extracellular matrix and further destroy the stability of atherosclerotic plaques [92]. Specifically, the senescent cells in the lesion showed expression of vital SASP cytokines and effector factors of inflammation, monocyte chemotaxis and proteolysis, including IL-1α, MCP-1, MMP-12 and MMP-13. This indicates that senescent cells can directly affect the development of core atherosclerosis through specific secretion factors [17]. For example, the SASP directly drives inflammation through IL-1α translocation to the cell surface and activates neighbouring VSMCs, ECs and macrophages, causing the spread of inflammation and promoting atherosclerosis by secondary pro-inflammatory cytokines [16]. Thus, removing senescent cells from advanced plaques prevents plaque enlargement and adaptive poor plaque remodelling processes connected with plaque instability, including thinning the fibrous cap and elastic fibre degeneration [17].

## Effect of the SASP on atherosclerosis via different senescent cells

**Fig. 3:**
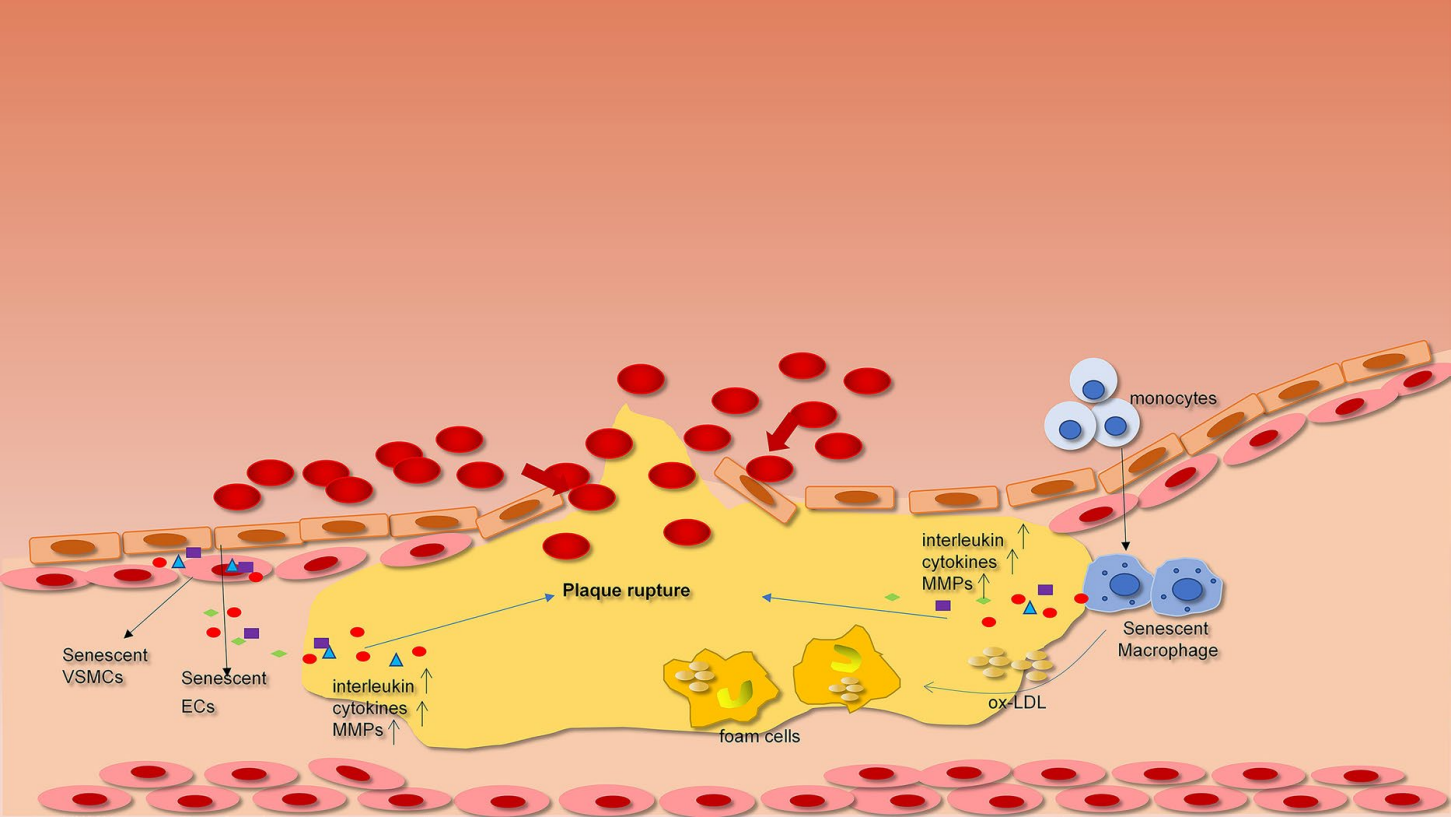
Various senescent cells contributions to the mechanisms of atherosclerosis

According to experiments with human samples and animal models, the accumulation of senescent cardiovascular cells plays an irreplaceable role in the promotion and development of cardiovascular ageing (Fig. 3) [20], aggravating the development of atherosclerosis and other cardiovascular and cerebrovascular diseases [20, 27]. The short-term accumulation of senescent cells in tissues is important to the stability of the internal environment and organ function, while the long-term accumulation of senescent cells in tissues leads to an imbalance of the internal environment and organ dysfunction. This reverse effect of cell senescence is due to the SASP [61], [93].

Furthermore, the paracrine signalling of the SASP has many negative influences including tissue remodelling, tumour generation and inflammatory ageing [94]. One of the most important mechanisms is inflammatory ageing. Inflammatory ageing contributes to the activation of leukocytes, ECs, and VSMCs, thereby accelerating vascular senescence and atherosclerosis due to chronic activation of inflammasomes and reduction of endogenous anti-inflammatory mechanisms [91]. We summarize a series of studies that suggest that different types of senescent cells play vital roles in the progression of atherosclerosis through the SASP factor (Table 1).

**Table 1:** Major SASP components in different senescent cell types contribute to the mechanisms of atherosclerosis

Cell type	SASP components	Effect on atherosclerosis	References
Senescent endothelial cell	IL-6	Form atherosclerotic plaques and cause thrombosis	[52]
	IL-1	Recruit more immune cells and form atherosclerotic plaques	[95]
	IL-8	Form atherosclerotic plaques and cause thrombosis	[52]
	IL-15	Form atherosclerotic plaques	[52]
	MCP-1	Recruit more immune cells and form atherosclerotic plaques	[95]
	TNF	Recruit more immune cells and form atherosclerotic plaques	[95]
	ICAM-1,VCAM-1	Recruit circulating monocytes and vascular injury	[17, [96]
	ET-1	Vascular endothelial dysfunction	[97]
	PAI-1	Cause thrombosis	[95, 98]
	VEGF	Promote plaque angiogenesis and vascular remodeling	[99, 100]
Senescent vascular smooth muscle cell	IL-1α	Leading to inflammation and monocyte chemotaxis, and proteolysis	[17]
	IL-6	Leading to inflammation and impaired vascular mitochondrial function	[45]
	IL-8	Induce angiogenesis in coronary plaques	[101]
	CCL-3, 4	Recruit monocyte/macrophage cells and increase plaque size	[101]
	MCP-1	Recruit monocyte cells	[17]
	TGF-β1	Enhance arterial stiffness and initiate the senescence of other cells	[102]
	MMP-1,2,3,7,8,9, 10,12,13,14	Fibrous cap of the plaque becomes thinner and extracellular matrix degradation	[103, 104]
	OPG	Vascular calcification;	[105]
	VEGF	Promote plaque angiogenesis and vascular remodeling	[99, 100]
Senescent T cell	IL-6	Leading to inflammation	[68]
	TNF-α	Leading to inflammation	[68, 106]
	IFN-γ	Induces the activation of macrophages	[107]
Senescent macrophage	IL-1β	Leading to inflammation	[11, 108]
	IL-6	Leading to inflammation	[11, 108]
	TNF-α	Leading to inflammation	[11, 109]
	MMP-3, 13	Extracellular matrix degradation and thinning of the fibrous cap in artery	[17, 110]

The “necrotic” core of late atherosclerotic plaques consists of lipids, foam cells, and debris. The fiber cap around the necrotic core is protective, which consists mainly of VSMCs. The stability of atherosclerotic plaques depends on the thickness of the fiber cap and the extent of inflammation of the fiber cap. At later stages, vascular cells such as VSMCs, ECs, monocytes and macrophages undergo senescence and release multiple cytokines as part of the SASP, which augment the preexisting inflammation.

## Senescence of endothelial cells

Endothelial cell senescence and endothelial cell dysfunction [111] are closely associated with the subsequent development and progression of CVD [52, 112, 113. And an increase in senescent ECs causes various diseases associated with arterial thrombosis [110. Endothelial cell senescence directly destroys the endothelial barrier by destroying cell proliferation, permeability, and motility, which may lead to endothelial cell erosion and intraplaque haemorrhage [9]. Additionally, there is evidence that ECs display strong SA β-gal activity, an important feature of senescent cells, in human coronary atherosclerotic lesions [13].

Endothelial cell senescence is associated with a significant increase in the production or release of a series of inflammatory factors and chemokines, known as the SASP [114]. Senescent ECs can produce more inflammatory factors such as interleukin-1, interleukin-6, interleukin-8, interleukin-15, MCP-1, TNF, and other mediators that promote atherosclerosis and thrombosis, and express cell adhesion molecules such as ICAM-1, and PAI-1 [52]. In addition, senescent ECs with the SASP express adhesion molecules including VCAM-1 and ICAM-1 and release many cell cytokines, causing the recruitment of monocytes. Then, circulating monocytes invade the subendothelial space, absorb ox-LDL and convert into foam cells [96]. Both foam cells and ECs with the SASP release excess chemotactic proteins, including interleukin-1, tumour necrosis factor, and MCP-1, which lead to the recruitment of immune cells and the formation of atherosclerotic plaques [95]. ECs with the SASP can lead to thrombosis via the activation of PAI-1, a known marker of senescence [95, 98]. Senescent HUVECs secrete many factors, such as interleukin-6, interleukin-8, and MCP-1, and growth factors such as VEGF, TGF and MMPs, compared with young cells [115].

Kotla S and his colleagues confirmed that D-flow-induced senescence and activation of ECs play an important role in promoting atherosclerosis. Their study found that TERF2IP-dependent gene expression and its role in D-flow-induced SASP Telomere Repeat-Binding Factor 2 (TERF2) Interacting Protein (TERF2IP) is a complex on the telomere that regulates the SASP [116]. Age-related vasculopathy, including atherosclerosis, is a major risk factor for vascular diseases (VDs). A chronic aseptic low-grade inflammatory state, also known as inflammation, is a feature of the elderly and is involved in the development of VD. It is becoming a vital mediator of inflammation and VD. This is because microRNA34a expression increases with vascular ageing, and it causes VSMCs and ECs to acquire the SASP [109]. It has been reported that senescent cells have this specific secretory phenotype that alters the homeostasis of surrounding tissues and the environment. For example, senescent endothelial cells and VSMCs have been shown to promote the expression of a variety of inflammatory factors, including IL-6 and IL-8. At the same time, senescent smooth muscle cells also release MMP-9 and generate less collagen, thus promoting vascular remodelling and atherosclerotic plaque instability [117].

## Senescence of vascular smooth muscle cells

VSMC senescence is closely related to a variety of cardiovascular diseases, including atherosclerosis, and has an important promoting effect [101, 118, 119]. Moreover, senescent VSMCs usually drive the instability of atherosclerotic plaques, leading to myocardial ischaemia and cerebral ischaemia [110. Senescence of VSMCs is associated with an enlarged necrotic core and plaque calcification. It has been reported that senescent VSMCs in atherosclerosis have the SASP, so they can not only promote inflammation by releasing inflammatory cytokines and chemokines in an interleukin-1-dependent way, but also recruit immune cell aggregation to accelerate the progression of atherosclerosis [102].

VSMCs from human atherosclerosis are characterized by high expression levels of genes associated with senescent cells, which explains why the cells have the SASP [12]. Senescent VSMCs acquire the phenotype secreted by osteoblasts and activate several osteogenic pathways such as osteoprotegerin (OPG), a key factor of the SASP and a CVD risk factor, which can promote plaque calcification [9]. Senescent VSMCs, together with SASP, lead to chronic vascular inflammation, loss of arterial function, and age-related conditions [102]. Senescent human VSMCs secrete a variety of SASP factors including interleukin-6, interleukin-8, and MCP-1, in an IL-1α-dependent manner, but the expression of anti-inflammatory factors, such as RANTES and IL-1R2, is decreased. Notably, the SASP molecules released by these senescent VSMCs can promote the recruitment of monocytes and macrophages.

Moreover, we found that senescent human vascular smooth muscle cells can actively promote the occurrence of atherosclerosis and plaque rupture by establishing the SASP. The SASP is characterized by sustained secretion of a variety of inflammatory factors and chemokines, which are regulated by the autocrine stimulation of senescent vascular smooth muscle cells by IL-1α. These powerful chemotactic signals lead to the aggregation of monocytes and lymphocytes, while secreted IL-1α leads to the activation of adjacent normal VSMCs and ECs, leading to pro-inflammatory cytokines secretion and amplification of adhesion receptor expression. Senescent VSMCs also release less collagen and more MMP-9, which degrades collagen in the matrix and weakens the plaque [101]. Consequently, VSMCs senescence not only promotes atherosclerotic progression, but also suppresses plaque repair.

Senescent VSMCs can promote chronic inflammation associated with atherosclerosis by an interleukin-1α-driven senescence-related secretory phenotype and initiating neighbouring cells into a pre-atherosclerotic state. These data indicate the need to block interleukin-1α to inhibit this potentially important chronic inflammatory source in atherosclerosis [101].

## Senescence of T cell

In addition to vascular system cells, senescent immune cells found in the walls of blood vessels also contribute to the development of atherosclerosis [79]. Senescent T cells secrete abundant pro-inflammatory factors such as tumour necrosis factor (TNF) and osteopontin, reminiscent of the SASP [106]. Lymphocytes (T cells) with shortened telomere length are more evident than myeloid cells in patients with coronary heart disease compared with age-matched controls, suggesting that T cells may play a vital role in ageing and telomere-mediated atherosclerosis [120].

The expression of CD27 and CD28 proteins is strongly downregulated in senescent T cells, while the secretion of pro-inflammatory factors such as IL-6 and TNF-α is increased, suggesting the presence of the SASP state [68]. Elevated levels of Th1 and Th17, cells and their cytokines interleukin-17, interleukin-21, and interleukin-23 are found in carotid atherosclerotic plaques, and these factors are associated with the promotion of atherosclerosis and plaque vulnerability [121]. Senescent T cells with CD4, CD28^null^, and CD45 re-expression are found to be a highly active subset that expressed increased tumour necrosis factor, INF-c, and CXCR-3. Moreover, many studies have shown that ageing affects memory T cells in unstable atherosclerotic plaques [79]. Meanwhile, CD4^+^ and CD8^+^ T-EMRA cells are considered to be predictors of cardiovascular-related mortality in elderly people [122].

In addition to the pro-inflammatory phenotype, T-EMRA cells show many characteristics of cellular senescence, such as decreased proliferation, mitochondrial dysfunction, increased secretion of tumour necrosis factor and interferon-γ, and increased p38 MAPK signaling [123]. Moreover, T-EMRA cells show atypical cytotoxic activity against plaque endothelial cells, potentially leading to plaque erosion [9].There is increasing evidence that senescent T cells, mainly cytotoxic CD8^+^ T cells, are also involved in the pathogenesis of CVD. In atherosclerosis and ACS, CD8^+^CD28^−^ T cell expansion is found in cohorts of CMV infected individuals and constitutes a risk factor for vascular dysfunction. Furthermore, during the development of atherosclerosis, CD8^+^ CD28^null^ CD27^−^ senescent T cells in inflammatory vascular walls continuously produce IFN-γ, a factor of the SASP, which activates macrophages to release MMPs to degrade the extracellular matrix [107]. Senescent T cells are present in peripheral blood mononuclear cells of humans [121]with atherosclerosis [124] and myocardial ischaemia [125].

## Senescence of macrophage

Some studies have shown that senescent immune cells show characteristics of the SASP, such as T cells [123, 126, 127], B cells [128] and macrophages [129]. The pro-inflammatory SASP properties of immune cells, particularly macrophages, appear to play an important role in maintaining low-grade chronic defect states in tissues [126]. Macrophages are the main senescent cells present with high levels of SA-β-Gal staining and the production of the SASP [17]. At the late stage of macrophage senescence, the net effect is the expression of the SASP, which aggravates the senescence of macrophages and releases outside the cells to impair the function of the surrounding cells. This process is known as “paracrine senescence” [53], which can cause more extensive inflammation [108]. Moreover, lncRNAs induce the SASP expression, that is, the upregulation of matrix metalloproteinase, interleukin-1β, interleukin-6 and TNF-α, and the down expression of IL-4 and IL-10, leading to inflammation, which is one of the signs of senescent macrophages [11, 108].

The involvement of macrophages in senescence and chronic inflammatory diseases is known as “macrophage senescence”. This concept highlights the critical role of the innate immune system, in particular pro-inflammatory macrophages as promotors and enhancers of premature senescence and in chronic diseases including CVD, and a recent study on the presence of pro-inflammatory M1 macrophages in plaque atherosclerosis leading to ACS [121]. Proatherogenic inflammatory factors including IL-6, IL-12, and reactive oxygen species (ROS), can promote the oxidative stress response of plaques [121]. Alternatively, studies have shown that senescent CD14^+^ CD16^+^ monocytes are capable of increasing inflammatory responses and interactions with endothelial cells. Thus, the accumulation of monocytes in senescence can promote the progression of chronic inflammation-related diseases including atherosclerosis [130]. In advanced atherosclerotic plaques, senescent macrophages work by increasing the formation of MMP-3 and MMP-13 [17], causing unstable features of atherosclerotic plaques including elastic fibre defects and thinning of the fibrous cap in the artery and the aorta [17, 110].

It has been reported that senescent macrophages formed in a BRD4-dependent epigenetic way are one of the crucial factors that drive the development of atherosclerosis. The chromosomal redistribution of BRD4 promotes the expression of senescence-associated proteins and the SASP genes regulated by NF-κB activation. Inhibition of BRD4 by inhibitors including siBRD4, JQ-1 or I-BET762 prevents the senescence of macrophages and lipid accumulation by reducing the expression of the SASP proteins in autocrine and paracrine senescence. This study is important for understanding the pathophysiology of age-related diseases and can guide future research on targeted drug treatment in the clinic [129].

## Effect of the SASP on atherosclerosis through immune dysfunction

### Immunosenescence and inflammation

**Fig. 4:**
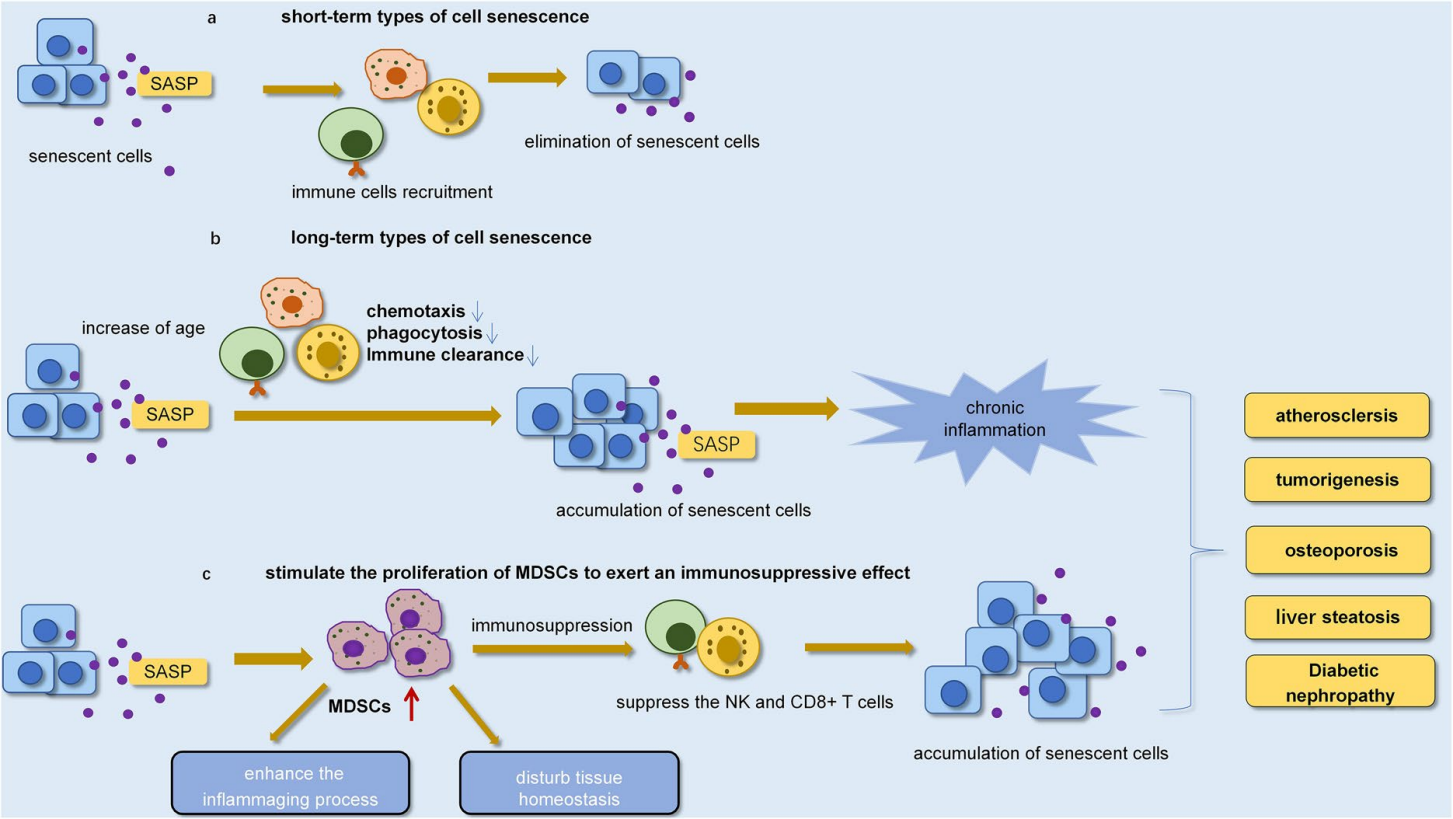
The effect of the SASP factors on the immune response. **a** Effects of the SASP factors secreted by short-term types of cell senescence on immune cells. **b** Effects of the SASP factors secreted by long-term types of cell senescence on immune cells. **c** The SASP plays an immunosuppressive role by promoting the proliferation of MDSCs

Whether cell senescence is good or bad depends largely on the time, short-term types seem to be beneficial because the immune system can efficiently clear away senescent cells (Fig. 4) [25]. A healthy immune system is absolutely necessary to clear senescent cells [131]. Senescent cells can efficiently mobilize immune cells to cause their own clearance, but clearance is delayed or impaired with age [11, 132–134]. It has been reported that the SASP has some benefits for tissue remodelling and repair [32], and can stimulate the immune system to clear away senescent cells. However, this protective process may eventually become less efficient with age and lead to increased inflammatory responses [79] and age-related diseases including atherosclerosis.

Immunosenescence is a phenomenon in which the immune system gradually declines with age [68]. When the chemotaxis and targeting of immune cells such as NK cells, macrophages and T cells to the increased number of senescent cells are reduced [24], age-related phagocytosis of neutrophils and macrophages is impaired, leading to the accumulation of senescent cells [135]. This finally leads to increased generation of the SASP, which promotes inflammation and the progression of age-related diseases [7]. Notably, high levels of pro-inflammatory factors promote the progression of inflammation, increase endothelial damage, and vascular remodelling damage, and ultimately lead to atherosclerosis [92, 121]. Immunosenescence is associated with chronic low-grade inflammation [136]. Immune monitoring decreases with age, for example, “older” T cells release more pro-inflammatory factors, including interleukin and tumor necrosis factor than “younger” T cells [137]. The accumulation of increasingly inefficient ageing-associated T cells may explain the susceptibility to fatal infections as well as the progression of noncommunicable diseases, including cancer or arteriosclerosis [106]. The major pathological processes of age-associated cardiovascular diseases, including endothelial dysfunction and arteriosclerosis, are primarily associated with immunosenescence and inflammation and can lead to atherosclerosis [138].

Notably, immunosenescence and the SASP are found in the elderly and in many age-associated diseases, including cardiovascular diseases as well as atherosclerosis, metabolic diseases, tumour promotion, and neurodegenerative diseases [121]. Chronic low-grade inflammation and immune senescence promote the development of multiple diseases mentioned above, so research on immune senescence could bring feasible solutions to the prevention and treatment of age-related diseases. Mitigating inflammation to inhibit the occurrence of age-associated diseases and the decrease in the immune response should be the focus of future research [106]. And attempts are being made to unite different strategies, such as anti-inflammatory drugs and immune checkpoint inhibitors, to optimally block inflammation [139]. For example, studies have shown that long-term and low-dose resveratrol reversed immune ageing and inflammation in elderly mice because it improves age-related elevations of 8-hydroxy-2′-deoxyguanosine, a label of oxidative DNA impairment [140].

### Immunosuppressive and immunosuppressive cells

Senescence cells through the SASP induce immune cell senescence and dysfunction, leading to excessive accumulation of senescent cells [110]. This is the immunosuppressive function of the SASP [141, 142]. An increase in senescent cells and the SASP cytokines can chronically damage the resident tissues of senescent cells and inhibit the function of immune cells [24]. SASP-related secretions include some chemokines that induce bone marrow-derived inhibitory cells (MDSCs) by promoting the proliferation of bone marrow [143]. MDSCs are the main immunosuppressive cells that suppress the immunological response [144, 145].

Studies have shown that Tregs and MDSCs release immunosuppressive factors, including interleukin-10 and TGF-b, which may inhibit the activation of T cells through membrane-bound PD-1 or PD-L1 connections [143, 146]. There is also ample evidence that immunosuppressive cells, such as MDSCs and Tregs, suppress the immunomodulatory ability and cytotoxic activity of NK and CD8 + T cells [147–149]. Thereby allowing senescent cells, including immune cells and nonimmune cells, to accumulate in the tissue during senescence. CD8^+^ CTLs and CD4^+^ Th1-like cells produce cytotoxic pro-inflammatory cytokines, including IFN-γ, and Th2-like cells produce IL-4 and TGF-β may be responsible for the elimination of many senescent somatic cells [107]. Overall, according to the above studies as indicated that, MDSCs may have an important role in the production of an immunosuppressive environment in chronic inflammatory illness.

The SASP of senescent cells induces an inflammatory state, activating immunosuppression. Immunosuppressive cells inhibit the monitoring and removal of senescent cells, thus forming a feed-forward loop between cell senescence and immune suppression, which not only contributes to the senescence progression itself but also promotes the development of age-associated illness. There is a large amount of evidence that some age-associated illnesses are related to the immunosuppression of MDSCs, such as atherosclerosis, liver steatosis, osteoporosis and type 2 diabetic nephropathy [150]. MDSC-induced immunosuppression weakens the clearance of senescent cells and interferes with energy metabolism and the production of tissue proteins [143]. The cooperation between senescent cells and immunosuppressed MDSCs regulates chronic inflammatory illness and promotes inflammation during senescence [143]. The discovery and study of therapies to remove senescent cells seem to be vital to improve the function of the immune system in elderly individuals. Many approaches are currently found to promote the immune removal of the abundant senescent cell burden in elderly individuals and age-associated diseases.

### Effect of the SASP on information transmission between senescent cells

In the vascular system, senescent cells may influence the effect and phenotype of surrounding cells in a variety of ways [151]. Senescent cells can communicate by direct contact between cells [152, 153], and cell fusion [154], by forming cytoplasmic bridges [155], through extracellular vesicle (EV) signal transduction [156] and through the SASP [57]. The SASP state occurs in senescent cells, and may maintain and amplify senescence via autocrine and paracrine regulatory loops. It can not only exacerbate its own SASP, but also induce the SASP in surrounding cells [91].

The SASP enables senescent vascular cells to send messages to surrounding cells and the microenvironment, influencing cell senescence, tissue regeneration, and embryonic development of neighbouring cells [20, 157]. Endothelial cells are connected through gaps, so the signals for inducing senescence can be sent between cells. The release of the SASP factors may promote paracrine senescence. For example, TGF-β family members, VEGF, and chemokines including CCL2 and CL20 may send senescence to normal adjacent cells [53], thus affecting their functions. Through the above mechanisms, senescent cells can lead to endothelial damage, flawed barrier function, elevated pro-inflammatory states, and pathological remodelling of the vessels during senescence [151], and then promote the occurrence of atherosclerosis. In addition, interactions between monocytes and endothelial cells are well-known to play an important role in inflammatory atherosclerosis. There is evidence that TNF-induced endothelial cells can generate more EVs composed of chemotaxis mediators, including ICAM-1, CXCL-10 and CCL-5, which have been taken up by monocytes and can benefit the expression of pro-inflammatory markers such as interleukin-6, and interleukin-8 and their adhesion and movement [158].

**Fig. 5:**
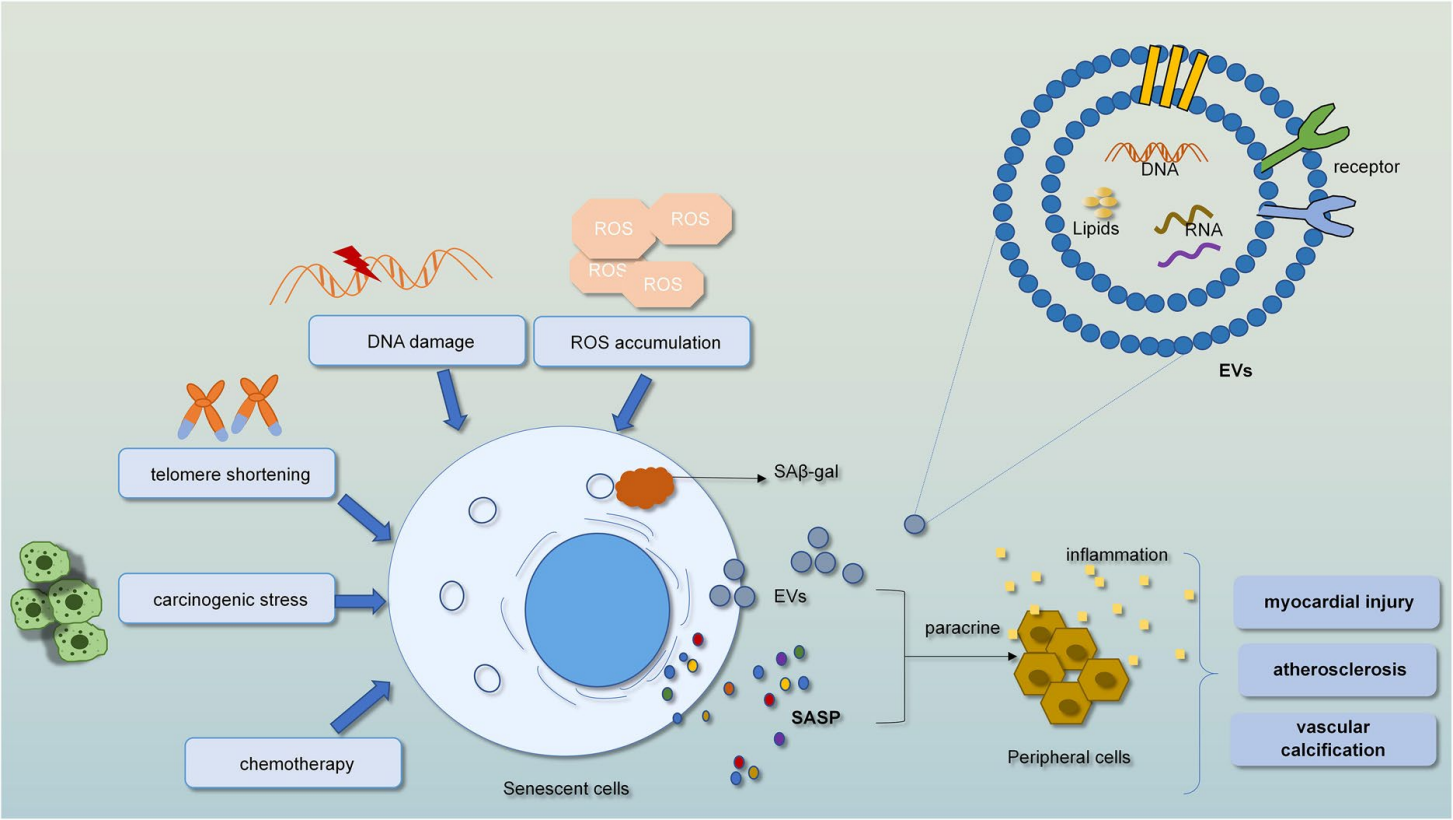
The SASP is an important mediator of the pathophysiological functions of senescent cells

The SASP is a method of cellular communication during senescence. The classical SASP consists of soluble cytokines, growth factors and ECM remoulding enzymes. However, the emerging SASP factors and other ways of what we call nonclassical intercellular communication have also been described during senescence [159]. Recently, comprehensive quantitative protein omics characterization of the SASP has revealed additional and various SASP factors that are secreted as soluble factors and exosomes, some of which have been indicated to be abundant in the plasma of humans during senescence and age-related illness. Moreover, exosomes were recently thought to be vital mediators of the paracrine senescence effect of the SASP [32]. There are clear studies that ageing expands the number of exosomes in the blood circulation and tissues, and in particular, the release of immunosuppressed exosomes is increased from senescent cells [160]. When they spread to neighbouring cells, they can enhance immunosuppression within recipient immunocytes, for example, tissue residence and recruitment of immunocytes. This indicates that the increase in immunosuppression with age weakens the removal of senescent cells, and then enhances senescence progression in tissues [160]. Among the markers of ageing, the SASP, especially the EV signal related to the SASP, plays a vital role in the transmission of ageing signals in paracrine or endocrine ways (Fig. 5). EVs, various small vesicles with lipid bilayers, can be secreted from various cells and have been detected in almost all humoral fluids [161]. Their communication between cells and organs suggests their involvement in the transmission of senescence signals in age-related diseases. They have a damaged influence on downstream targets at the immune, inflammatory, gene expression and metabolic levels [61]. In age-related diseases, senescent cells play a vital role in the transmission of the SASP factors to adjacent cells and tissues, with different EVs being representative. CVDs are the most common age-related diseases that manifest as tissue, cell, molecular and functional changes of the heart with age. A large number of circulating premature ageing and anti-ageing mocules from various systems simultaneously coordinate the ageing progression of the cardiovascular system. Moreover, they may lead to vascular ageing, impaired angiogenesis and promote chronic inflammatory response, pathological remodelling, atherosclerosis, and myocardial damage [61].

Intrinsic and extrinsic factors contribute to cellular senescence. Then, senescent cells secrete pro-inflammatory cytokines as part of the SASP, this process leads to the increased inflammation in atherosclerotic plaques. Meanwhile, the SASP enables senescent cells to communicate with other cells and promotes senescence and inflammation via paracrine action, eventually lead to vascular-related diseases

For example, in nonalcoholic fatty liver disease and cardiovascular disease models, EVs can shuttle between the liver and the vasculature, indicating their role in long-distance interorgan communication. MiR-1-containing lipid hepatocyte-derived EVs inhibit KLF4 expression, while activating the NF-kappa B signalling pathway, thus participating in the development of atherosclerosis by promoting endothelial cell inflammation and senescence [162]. Age-related EVs significantly induce vascular calcification and are a hazardous factor for atherosclerosis and myocardial damage. EVs obtained from senescent ECs and plasma of many old patients contain high levels of Annexin, bone morphogenetic protein-2 and Ca2 + , which can promote the progression and proliferation of calcified aortic SMCs in humans. Monocyte-endothelial cell interactions also play an important role in inflammatory atherosclerosis [61]. EV-derived miRNAs from atherogenic macrophages, especially miR-146a, can promote the progression of atherosclerosis by decreasing cell removal and supporting macrophage entrapment in the arterial wall [163].

### Treatment of targeted senescent cells

Antiaging interventions should attract more attention because of the limited efficacy in treating atherosclerosis, cancer, and neurodegenerative diseases. The use of senolytics offers an alternative antiaging approach that offers major health benefits. In animal models of atherosclerosis, many antiaging drugs are promising candidates for improving human health because reducing senescent cells can delay ageing and delay the development of chronic diseases [164]. Multiple drugs and molecules play an anti-aging role in atherosclerosis (Table 2). In principle, sensory therapy methods may be extensively divided into two parts: the senolytic strategy (an antiaging strategy that selectively removes senescent cells) and the senomorphic strategy (an antiaging strategy that inhibits the SASP without affecting cell death).

**Table 2:** Anti-aging effects of multiple drugs and molecules in atherosclerosis

Drugs or molecules	Effects	References
Quercetin	Reduce lipid accumulation and senescence phenotype	[165]
Metformin	Inhibiting the inflammation, oxidative stress, foam cell formation and apoptosis of macrophages	[166]
GCV	decreased expression of MMPs linked to plaque destabilization	[17]
Statins	Accelerating DNA damage repair; suppressing oxidative stress	[59, 167]
Bradykinin	Protects ECs from superoxide-induced senescence	[168]
Fisetin	Regulating lipid metabolism; anti-aging; anti-oxidation; anti-inflammatory	[169]
ABT-263	Eliminate senescent cells	[17]
Dasatinib + quercetin (D + Q)	Reduces the number of senescent VSMCs; reduced aortic calcification	[170]
Resveratrol	Prevent ROS-induced senescence in vascular cells	[114]
Nicotinamide mononucleotide	Reduce the NAD depletion; reduce vascular aging	[171]
Ethanol	Resisting endothelial senescence and the SASP inflammatory factor secretion	[172]
Sirtuin 6	Preserved telomere integrity; delayed cellular senescence; reduced inflammatory cytokine expression	[173]
TRF2	Regulate VSMCs senescence; increased the fibrous cap area; reduced core size	[174]
Pin1	rescued cellular senescence in atherosclerotic VSMCs	[175]

### Anti-atherosclerosis by targeting senescent cells

Large numbers of SAβG-positive vascular smooth muscle cells, endothelial cells and macrophages are found in aged arteries and plaques in atherosclerosis compared with surrounding young cells and normal cells, indicating that atherosclerosis connects with premature cell senescence. Cellular senescence promotes many stages of the progression of atherosclerosis. It is a key driver of atherosclerosis formation and maturation, with evidence suggesting that selective removal of these cells by drugs is promising for the treatment of atherosclerosis [17]. Strong preclinical studies indicate that removing p16ink4a expressing senescent cells can significantly extend lifespan in mouse models [151]. Senescent cells can badly influence adjacent cells by altered secretion phenotypes: the SASP. The SASP promotes senescence in normal cells and causes the development and progression of age-associated disorders, including atherosclerosis. Thus, eliminating senescent cells may delay age-related degeneration and prolong life [176].

Senescent cells are a vital source of local, low-grade chronic inflammation and plaque instability. Therefore, it is also a promising upstream treatment target. Targeting senescent cells or the progression of senescence has been explored to improve atherosclerosis, reduce inflammation, and enhance plaque stability [9]. Selective removal of P16INK4A-active senescent cells in transgenic animal models of accelerated ageing (INKATTAC and P16-3MR mice) extended the healthy period [19, 177, 178]. Moreover, the removal of senescent cells in versions of this genetic model or therapy mouse with special senolytics can extend the health span [179, 180], restore vascular reactivity [170], and stabilize atherosclerotic plaques [17]. Senescent vascular cells not only acquire the SASP, but also exhibit the production of ROS. Pharmacological activators of Nrf2, such as resveratrol, can attenuate ROS-induced DNA damage and hope to inhibit ROS-induced senescence in cardiovascular cells [151]. Emerging studies ablating senescent cells using genetic systems or drugs suggest that eliminating senescent cells reduces inflammation within tissues [19, 181]. Many initial studies indicate that senolytic medicine can suppress atherogenesis such as dasatinib, the polyphenols quercetin and fisetin, and the Bcl-2 or Bcl-XL inhibitor navitoclax (ABT263). They can efficiently and selectively remove senescent cells from the vascular system [17, 151].

A study indicates that phosphate binder prevents phosphate-induced cell senescence of VSMCs and vascular calcification in a modified [182], and Sirtuin 1 inhibits hyperphosphatemia-induced calcification of VSMCs [183]. It has been reported that macrophage telomerase reverse transcriptase (TERT) damage leads to a senescent phenotype [184], and that TERT is overexpressed in atherosclerotic plaques. Thus, the induction of TERT expression can inhibit the senescence of macrophages and may play a vital anti-atherosclerosis role. In addition, some of the currently available drugs and compounds, including antioxidants, statins, ACE inhibitors and ARBs, may delay premature ageing and delay atherosclerosis by changing ROS and oxidative DNA damage [5]. Although anti-aging treatments are expected to treat age-associated illnesses including atherosclerosis, many difficulties remain to be solved [119]. Mouse experiments have shown that the removal of senescent cells can delay the onset and development of atherosclerosis and may also delay the development of established plaques [17]. However, whether they are sufficient to reverse the atherosclerotic plaque developed in humans remains to be studied [119].

### Anti-atherosclerosis by inhibiting the regulatory pathway of the SASP

Senomorphic treatment inhibits the pro-inflammatory properties of senescent cells and normalizes the process of the SASP in the ageing environment by regulating many biochemical pathways such as m-TOR, p38-MAPK, NF-kappa B, JAK/STAT, ROCK and glucocorticoid receptors [61]. The component of the SASP varies depending on the cell types of the senescent cells, the special promoters that led to senescence and the hormonal milieu. Therefore, hormones and medicines, including rapamycin, metformin, JAK1/JAK2 inhibitors and glucocorticoids may inhibit the SASP [8].

For example, rapamycin may inhibit the SASP from senescent fibroblasts, possibly by inhibiting m-Tor-dependent IL-1α mRNA translation and the JAK/STAT3 pathway [185]. In addition to having anti-inflammatory properties, rapamycin can inhibit the m-TOR pathway [186] to play an antiaging role [187]. Meanwhile, many inhibitors of NF-κB, including BMS-06 and vinpocetine, have been shown to inhibit the progression of atherosclerosis in mice [188]. Emerging experimental evidence indicates that p38 MAPK inhibitors inhibit the SASP, protect cells from senescence and inhibit senescent paracrine signalling in vitro [189]. Statins are effective medicines to delay age-related inflammation in the arterial tube wall and slow the progression of atherosclerosis. It has been shown that statins play a role in delaying endothelial senescence and T cell senescence by p38-mediated SASP inhibition and cell cycle regulation [190]. Specific agents can also directly target the mechanisms of DNA damage and DDR to reduce atherosclerosis. Chloroquine plays a role in slowing atherosclerosis through p53-dependent ATM signalling in animal models [5].

We found that stress-induced DNA damage mainly caused the SASP through the formation of CCFs. Of interest, we also suggest that metformin and rapamycin-induced autophagy activation usefully reduced the number and level of CCFs and inhibited the activation of the cGAS-sting-NF-κB-SASP cascade and cell senescence. These effects of autophagy activation factors explained that autophagic lysosomal function contributed to the removal of CCF and the inhibition of the SASP, further supporting the role of the lysosomal inhibitor buffamycin A1 in blocking autophagy-mediated CCF clearance and ageing inhibition [191]. Moreover, metformin has been reported to improve the inflammaging profile of TH17 cells by enhancing T cell autophagy in vitro and in humans and improving the biological function of mitochondria [192].

### Anti-atherosclerosis via extracellular vesicles (EVs)

There is increasing evidence that EVs released by senescent cells have special characteristics and help regulate the phenotype of receptor cells, similar to SASP-like factors. Therefore, EVs released by senescent cells, that is, senescence-associated EVs, appear to be a special SASP factor [193]. In the senescence microenvironment, senescent cells secrete EVs that can transmit senescence signals in an amplified manner. Moreover, they can be cell-free therapeutic tools. Not only did they have no risk of inducing tumours, they also showed a lower immune rejection. EVs are emerging candidates for anti-ageing treatments [61].

Therapeutic EVs come from natural, modified or artificial sources. EVs derived from stem cells contain a variety of substances that can perform antioxidant stress and anti-inflammatory activities and ultimately achieve anti-ageing effects. Emerging evidence suggests that EVs donated by centenarians can have many powerful antioxidants to maintain cell, tissue, and system balance. In fibroblasts of centenarians, EVs include high levels of the enzyme RNAseH2C, which can prevent the DNA damage-induced inflammatory response and decrease the age-related chronic inflammatory response [194]. Senescent endothelial cells can damage the integrity of the vascular endothelium, leading to vascular ageing and a series of disorders, including atherosclerosis. In vitro experiments showed that MSC-sEV could reduce the ageing biomarkers of oxidative stress-induced senescent ECs, reduce the SASP, and rescue angiogenesis, migration and other dysfunction in oxidative stress-induced senescent endothelial cells [195].

EVs take an active part in every stage of the progression of atherosclerosis, and contribute to the formation of thrombi which can lead to a major adverse cardiovascular event. However, certain EVs can have protective biological functions in response to environmental and some stimuli [196]. For example, after treatment with calcium and phosphate, macrophages promote vascular calcification by releasing calcified MVs rich in S100A9 and conjunctin V. In contrast, exosomes from MSCs carrying SIRT6 can target VSMCs and prevent the calcification of blood vessels [196, 197]. EC-derived EVs inhibit the activation of monocytes by delivering miR-10a and targeting the NF-kappa B pathway. It is evidence that injecting this EV into mice can inhibit the activation of monocytes [198].

## Conclusion

Various senescent cells can develop the SASP, which can change the cell microenvironment and produce a chronic inflammatory response, causing tissue damage during ageing. Furthermore, the SASP is also a vital factor for the occurrence and development of multiple ageing-related diseases, particularly atherosclerosis. Therefore, the prevention of accelerated cellular senescence and the SASP represents an important therapeutic opportunity, and understanding the mechanisms responsible for this change is essential for the promotion of prevention and therapy of atherosclerosis and other age-associated diseases. In this review, we have highlighted some potential strategies that can mitigate the detrimental effects of senescent cells and the SASP. Although there are many unresolved difficulties and challenges, we suggest that more experiments are needed to study the real nature of the role played by the SASP in cellular senescence and atherosclerosis. In the future, we believe that more studies will be conducted to develop effective treatments and identify novel potential therapeutic targets for ageing-related diseases, especially atherosclerosis, in an ageing population.

## Data Availability

Not applicable.

## Abbreviations

SASP: Senescence-associated secretory phenotype
CVD: Cardiovascular disease
LDL: Low-density lipoprotein
SaβG: Senescence-associated beta galactosidase
VEGF: Vascular endothelial growth factor
PDGF: Platelet derived growth factor
MMPs: Metalloproteases
EV: Extracellular vesicle
TNF: Tumor necrosis factor
ECM: Extracellular matrix
DAMPs: Damage-associated molecular patterns
M-TOR: Mammalian target of rapamycin
DDR: DNA damage reaction
CCFs: Cytoplasmic chromatin fragments
VSMCs: Vascular smooth muscle cells
ECs: Endothelial cells
ICAM-1: Intercellular adhesion molecule-1
VCAM-1: Vascular cell adhesion molecule-1
ET-1: Endothelin-1
ROS: Reactive oxygen species
HUVEC: Human umbilical vein endothelial cells
OPG: Osteoprotegerin
TERF 2: Telomere repeat-binding factor 2
MDSCs: Marrow-derived inhibitory cells
TERT: Telomerase reverse transcriptase
ACS: Acute coronary syndrome
VDs: Vascular diseases
